# Loneliness Relates to Functional Mobility in Older Adults with Type 2 Diabetes: The Look AHEAD Study

**DOI:** 10.1155/2020/7543702

**Published:** 2020-10-30

**Authors:** Jeanne M. McCaffery, Andrea Anderson, Mace Coday, Mark A. Espeland, Amy A. Gorin, Karen C. Johnson, William C. Knowler, Candice A. Myers, W. Jack Rejeski, Helmut O. Steinberg, Andrew Steptoe, Rena R. Wing

**Affiliations:** ^1^Department of Allied Health Sciences, University of Connecticut, Storrs, CT, USA; ^2^Department of Biostatistics and Data Science, Wake Forest School of Medicine, Winston-Salem, NC, USA; ^3^Department of Preventive Medicine, University of Tennessee Health Science Center, Memphis, TN, USA; ^4^Institute for Collaboration on Health, Intervention and Policy, and Psychological Sciences, University of Connecticut, Storrs, CT, USA; ^5^Diabetes Epidemiology and Clinical Research Section, Phoenix Epidemiology and Clinical Research Branch, National Institute for Diabetes, Digestive and Kidney Diseases, Phoenix, AZ, USA; ^6^Pennington Biomedical Research Center Baton Rouge, Baton Rouge, LA, USA; ^7^Department of Health and Exercise Science, Wake Forest School of Medicine, Winston-Salem, NC, USA; ^8^Department of Endocrinology, University of Tennessee Health Science Center, Memphis, TN, USA; ^9^Behavioural Science and Health Institute of Epidemiology & Health, University College London, London, UK; ^10^Department of Psychiatry and Human Behavior, The Miriam Hospital and Alpert School of Medicine at Brown University, Providence, RI, USA

## Abstract

**Objective:**

Little is known about the impact of loneliness on physical health among elderly individuals with diabetes. Here, we examined the relationship of loneliness with disability, objective physical function, and other health outcomes in older individuals with type 2 diabetes and overweight or obesity.

**Method:**

Data are drawn from the Look AHEAD study, a diverse cohort of individuals (ages 61–92) with overweight or obesity and type 2 diabetes measured 5–6 years after a 10-year weight loss randomized, controlled trial.

**Results:**

Loneliness scores were significantly associated with greater disability symptoms and slower 4-meter gait speed (*ps* < 0.01). Loneliness did not differ across treatment arms. *Discussion*. Overall, these results extend prior findings relating loneliness to disability and decreased mobility to older individuals with type 2 diabetes and overweight or obesity.

## 1. Introduction

Loneliness is a subjective state, reflecting a lack of desired closeness with friends, family, and loved ones. Compared with structural measures of social contacts, counting an individual's opportunities for interaction with other people, loneliness assesses the function of social interactions in allowing a person to feel connected to others [1]. Living alone, widowhood, poor health status, and poor functional status each increase risk for loneliness [2, 3]. Roughly, 25–43% of adults over the age of 70 report being lonely [4].

Loneliness is a well-established correlate of mental health, quality of life [5–7], and early mortality in older adults [8–14]. Moreover, loneliness has previously been shown to relate to disability [15, 16] and impaired mobility [17]. For example, loneliness predicted a faster rate of objectively-measured motor decline, defined by motor function and muscle strength, over five years of follow-up among 985 men and women, with a mean age of 80 [17]. Perissinotto and colleagues [13] found that loneliness was related to greater difficulty with activities of daily living and mobility at six year follow-up among over 14,000 men and women over the age of 60 in the Health and Retirement Study. Higher levels of loneliness also predicted frailty as defined by the Fried Formula at 4 year follow-up among 2,817 individuals over 60 years of age from the English Longitudinal Study of Aging [15]. Interestingly, Hoogendijk and colleagues [18] reported that frailty increased the risk for loneliness over 3 years, suggesting that the relationship between loneliness and physical function may be bidirectional [18].

Little is known about how loneliness relates to health status among older individuals with type 2 diabetes. In the United States, 25% of individuals over the age of 65 have type 2 diabetes [19] increasing the risk for early mortality, cardiovascular disease, renal disease, dementia, functional impairment, depression, and vision impairment [20]. Although less stringent treatment goals can be recommended for elderly individuals, the need for diabetes self-management remains including treatment adherence, nutrition, and exercise [21]. Social support improves diabetes self-management, medication adherence, diet change, active lifestyles and, in some cases, glycemic control [22]. Conversely, loneliness is associated with less physical activity [23, 24] and poorer sleep quality [25, 26]. As such, it is plausible that loneliness may relate to health outcomes among elderly individuals with type 2 diabetes but these associations have not been established.

Look AHEAD was a randomized controlled trial designed to determine whether 10 years of intensive lifestyle intervention (ILI), comprised of calorie restriction and physical activity promotion to achieve weight loss, improves health outcomes among older individuals with type 2 diabetes and overweight or obesity, relative to a Diabetes Support and Education (DSE) control group. The cohort was reassessed for aging-related outcomes at 15-year follow-up, including loneliness measured for the first time. The goal of this paper is to characterize the prevalence of loneliness among individuals with type 2 diabetes and overweight or obesity in the Look AHEAD cohort and to determine cross-sectional associations of loneliness score with self-reported disability and objective mobility and other health indicators, including HbA1c, quality of life, and depressive symptoms. It is hypothesized that loneliness will relate to (1) greater disability and decreased mobility and physical function, as defined by the 400 m walk, grip strength, and the Short Physical Performance Battery and (2) higher HbA1c and depressive symptoms and lower quality of life.

## 2. Methods

### 2.1. Research Design

Look AHEAD is a randomized, controlled trial designed to test whether 10 years of ILI, combining calorie restriction and physical activity to produce weight loss, improves health outcomes among individuals with type 2 diabetes and overweight or obesity, relative to DSE [27, 28] (see Supplementary File 2). The cohort was reassessed at year 15 to continue to follow diabetes and aging-related outcomes, including measuring loneliness for the first time. The study enrolled 5,145 men and women, aged 45–76 at baseline. The present study is cross-sectional and derives variables from 15 year follow-up when participants had a mean age of 75 (range: 61–92). All Look AHEAD participants who were attending clinical visits were included (*n* = 3187). Look AHEAD participants who were followed only through telephone interviews (*n* = 300) were excluded because loneliness was not queried.

### 2.2. Study Interventions

Eligible patients were randomly assigned to participate in ILI (intervention group) or DSE (comparison group), with stratification according to clinical site. Curricula for the two study groups were developed centrally and have been described in detail previously [27, 28].

### 2.3. Intensive Lifestyle Intervention (ILI)

The ILI included calorie restriction, low-fat diet, and increased physical activity and was designed to induce at least a 7% weight loss at year 1 and to maintain this weight loss in subsequent years. ILI participants were assigned a calorie goal (1200–1800 kcal/d based on initial weight), with less than 30% of total calories from fat (<10% from saturated fat) and a minimum of 15% of total calories from protein. The exercise goal was at least 175 minutes of physical activity per week, using activities similar in intensity to brisk walking. ILI participants were seen for 3 groups and one individual session per month for the first 6 months and 2 group, one individual session per month for the next 6 months, and at least monthly through year 10. ILI was effective in inducing and sustaining weight losses relative to the control condition throughout follow-up [28].

### 2.4. Diabetes Support and Education (DSE)

DSE featured three group sessions per year focused on diet, exercise, and social support during years 1 through 4. In subsequent years, the frequency was reduced to one session annually.

## 3. Measures

### 3.1. Loneliness

Loneliness was measured using the UCLA Brief Loneliness Scale [29]. The scale contains three questions: “How often do you feel that you lack companionship?”, “How often do you feel left out?”, and “How often do you feel isolated from others?” Each item has the response choices of “Hardly ever,” “Some of the time,” and “Often,” assigned scores 0, 1, and 2 respectively. These scores for each of the items are summed to give a total score. The prevalence of loneliness has also been defined as reporting “Some of the time” or “Often” relative to “Hardly ever” for at least one of the three questions: “How often do you feel that you lack companionship?”, “How often do you feel left out?” and “How often do you feel isolated from others?” [13]. The UCLA Brief Loneliness Scale was shown to have a strong correlation with the full UCLA Loneliness Scale (*r* = 0.82) and to have reasonable internal consistency (Cronbach's *α* = 0.72) [29].

### 3.2. Disability and Physical Function

#### 3.2.1. Pepper Assessment Tool for Disability (PAT-D)

The PAT-D is an 18-item self-report questionnaire designed to assess disability in older adults. Participants are asked to rate: “How much difficulty, if any, do you have with each of these activities? Think about the past month. How hard was it to do the activity because of your health?” Items include questions such as “Moving in and out of bed” and “Dressing yourself.” Responses range from “Usually did with no difficulty” (1) to “Unable to do” (5) with the possibility of endorsing “Usually did not do for other reasons.” Scores are averaged across the 18 items. The PAT-D has shown strong internal consistency (*α* = 0.82) and test-retest reliability (*r* > 0.70).

#### 3.2.2. Physical Function Tests

Objective physical function was assessed in the full cohort at an average of 15–16 year follow-up. The Short Physical Performance Battery Expanded (SPPBexp) [30], a modestly expanded form of the Short Physical Performance Battery [31] designed to minimize ceiling effects of the SPPB when used in well-functioning populations, was administered to assess lower extremity physical function. The SPPB consists of standing balance tasks (side-by-side, semi- and full-tandem stands for 10 seconds each), a 4 m walk to assess usual gait speed and time to complete five repeated chair stands. The SPPBexp increased the holding time of the standing balance tasks to 30 seconds and added a single leg stand. The SPPBexp component scores are calculated as the ratio of observed performance to the best possible performance and summed to provide a continuous score ranging from 0 to 3, with higher scores indicative of better performance. Usual walking speed over 20 m and walking endurance over 400 m were measured [32]. The course was 20 m long and marked by cones at each end. Participants were instructed to walk at their usual pace, and time to complete the first 20 m and the longer 400 m was recorded. Grip strength (kg) was measured twice in each hand using an isometric Hydraulic Hand Dynamometer (Jamar, Bolingbrook, IL). The maximum force from two trials for the stronger hand was used in the analyses.

### 3.3. Other Health Indicators

#### 3.3.1. Personal Health Questionnaire-9 (PHQ-9)

The PHQ-9 is a self-administered questionnaire assessing depressed mood and depression severity [33]. The questionnaire asks “How often, over the past two weeks, have you been bothered by any of the following problems?” for nine questions, including “Little interest or pleasure in doing things” and “Feeling down, depressed, or hopeless.” Response options include “Not at all,” “Several days,” “More than half of the days,” and “Nearly every day.” Depressed mood severity was calculated by assigning response options scores of 0–3 based on increasing frequency and summing the scores (range: 0–27). The PHQ-9 has strong internal consistency (*α* = 0.89) and test-retest reliability (*r* = 0.84) in clinical samples [33]. The PHQ-9 does not include a loneliness item.

#### 3.3.2. Antidepressant Medications

Participants brought all prescription medications to their annual clinic assessment visits, and these medications (but not the dosages) were recorded by study staff. Antidepressant medications were identified using the Food and Drug Administration classification system.

#### 3.3.3. Quality of Life

Quality of life was assessed using the SF-36 General Health questionnaire [34]. The questionnaire asks participants: “In general, would you say your health is…” with responses ranging from Excellent (1) to Poor (5) on a 1–5 scale. Lower values indicate better general health.

#### 3.3.4. HbA1c and Diabetes Medications

HbA1c was assayed from fasting blood samples. Six major classes of diabetes medications were categorized from the Food and Drug Administration classification system and were used as covariates in analyses of HbA1c.

### 3.4. Statistical Analysis

Primary analyses were conducted using linear or logistic regression depending on the outcome. Model 1 tested the association of loneliness, age, sex, race, and ethnicity with the function- and health-related variables. Model 2 added depressive symptoms and antidepressant medications to determine whether loneliness relates to the other variables independent of correlated constructs also known to relate to health outcomes. For the relationship of loneliness to HbA1c, the six major categories of diabetes medications were added as covariates to Model 2. Treatment arm was added in Model 3 to determine whether loneliness differs by ILI.

PHQ-9 and PAT-D scales are extremely skewed, even after log transformation, and thus were dichotomized at their lowest value vs anything else.

## 4. Results

### 4.1. Descriptive Statistics

Descriptive statistics for baseline and the Look AHEAD E visit (15-year follow-up) are presented in Table 1. The balance afforded by the original randomization was maintained at the 15-year visit: no differences in baseline age, sex, race, or Hispanic ethnicity were observed. However, several health indices continued to show intervention effects, including lower BMI (32.9 vs 33.6; *p*=0.002), faster gait speed (4.85 vs 5.00 seconds; *p*=0.01), and less insulin use (43.4% vs 49.5%; *p*=0.0009) in the ILI compared with DSE groups. No differences in loneliness by Look AHEAD treatment arm were observed (*p*=0.11).

### 4.2. Prevalence of Loneliness

Thirty-eight percent of the Look AHEAD samples reported being lonely as defined by endorsing “Sometimes” to at least on the three questions on the UCLA Brief Loneliness Survey. Nine percent reported “Often” for at least one question.

### 4.3. Differences in Loneliness Scores by Demographics and BMI

As seen in Supplementary Table 1, levels of loneliness differed meaningfully by demographics and BMI. The mean loneliness score was higher in women compared to men (*p* < 0.0001), individuals of Black, Hispanic, or other races and ethnicities compared to whites (*p*=0.0001), individuals with less formal education (*p* < 0.0001), and individuals who have a BMI ≥ 40 kg/m^2^ at baseline (*p*=0.02). A BMI ≥ 40 kg/m^2^ at 15 year follow-up also was strongly related to loneliness (*p* < 0.0001), but no differences in loneliness by age were observed at 15 year follow-up (*p*=0.38).

### 4.4. Loneliness, Disability, and Mobility

In models adjusting for age, sex, race, and ethnicity (Table 2, Model 1), greater loneliness was significantly related to greater self-report of disability on the Pepper Disability Test (OR = 1.47; *p* < 0.0001), slower gait speed (*β* = 0.19 ± 0.02; *p* < 0.0001), and weaker hand grip (*β* = −0.39 ± 0.10; *p* < 0.0001). Loneliness did not correlate with the 400-meter walk test speed (*β* = 0.03 ± 0.03; *p*=0.2375).

After further adjustment for depression symptoms and antidepressant medication use (Table 2, Model 2), greater loneliness continued to significantly relate to higher Pepper Disability Test scores (OR = 1.24; *p*=0.0018) and slower gait (*β* = 0.10 ± 0.03; *p*=0.0003), but the association with grip strength was reduced to nonsignificance (*β* = −0.17 ± 0.12; *p*=0.1592). Again, these associations were not substantially altered by statistical control for treatment arm (Table 2, Model 3).

### 4.5. Loneliness, Depressive Symptoms, Antidepressant Medications, and Quality of Life

In models adjusting for age, sex, race, and ethnicity (Table 2, Model 1), greater loneliness was significantly related to higher PHQ-9 scores (OR = 1.89; *p* < 0.0001) and a greater likelihood of taking antidepressant medications (OR = 1.32; *p* < 0.0001). After further adjustment for antidepressant medication use (Table 2, Model 2), greater loneliness continued to relate to higher PHQ-9 scores (OR = 1.75; *p* < 0.0001). Similarly, after controlling for PHQ-9 scores, greater loneliness continued to relate to antidepressant use (OR = 1.12; *p* < 0.0001). These associations were not substantially altered by statistical control for treatment arm (Table 2, Model 3).

In models adjusting for age, sex, race, and ethnicity (Table 2, Model 1), greater loneliness was significantly related to lower self-rated general health on the SF-36 (*β* = 0.14 ± 0.01; *p* < 0.0001), and this association remained significant after controlling for depressive symptoms and antidepressant medication use (*β* = 0.03 ± 0.01; *p*=0.0063) (Table 2, Model 2).

### 4.6. Loneliness and HbA1c

Loneliness did not correlate with HbA1c in models adjusted for age, sex, race, and ethnicity (*β* = 0.03 ± 0.02; *p*=0.1297), nor with further adjustment for depressive symptoms, antidepressant medications, glucose-lowering medication, or treatment arm.

### 4.7. Interaction with Treatment Arm

Treatment arm interacted with loneliness in its association with two mobility measures: 400-meter walk test (*p*=0.03) and gait speed (*p*=0.03; Table 3). In each case, physical function was similar across the spectrum of loneliness scores in the ILI, whereas higher loneliness was related to poorer physical function in DSE. Supplementary Figures 1(a) and 1(b) illustrate the interactions with loneliness depicted at the 10^th^ (loneliness = 3) and 90^th^ (loneliness = 6) percentiles of loneliness score.

There was also an interaction of loneliness and treatment arm in their associations with HbA1c (*p*=0.04, Table 3). In those with lower loneliness scores, there was no differential effect between treatment arms. However, in those with higher loneliness scores, HbA1c was lower in the ILI compared to the DSE (Supplementary Figure 1(c)).

## 5. Discussion

In this sample of older individuals who have type 2 diabetes and overweight or obesity, loneliness was associated with higher disability scores and slower gait speed after statistical adjustment for several potential confounders. Loneliness was also associated with higher depressive symptoms and antidepressant medication use and poorer health-related quality of life after similar adjustment. These results identify loneliness as an important correlate of physical and mental health among aging individuals with an elevated body mass index and type 2 diabetes.

After adjustment for demographic variables, loneliness also correlated with a weaker hand grip but the association weakened to nonsignificance after further adjustment for depressive symptoms and use of antidepressant medications, indicating that loneliness is not independently related to hand grip strength in this study. Loneliness did not significantly relate to HbA1c or the 400-meter walk test in any of the models.

The prevalence of loneliness was similar to prior research in elderly individuals. Roughly 38% of these individuals with type 2 diabetes and obesity or overweight reported loneliness at least some of the time, and 9% endorsed experiencing at least one of the loneliness questions often. In the most direct comparison, Perissinotto and colleagues used the UCLA Brief Loneliness Scale (as was used in this paper) to estimate prevalence of loneliness in the Health and Retirement Study, including noninstitutionalized individuals over the age of 65 [13]. Forty-three percent endorsed loneliness on one of the questions at least some of the time, and 13% reported feeling lonely often, suggesting that the prevalence of loneliness in Look AHEAD is roughly comparable with the U.S. population over the age of 65.

Levels of loneliness did not differ by randomized treatment arm. This is likely due to the measurement of loneliness 15 years after the initiation of the intervention, and 5 years following the end of the intervention. More research is needed to determine the impact of lifestyle intervention for weight loss on loneliness, particularly in individuals with BMI ≥ 40, who showed an increased loneliness scores both at baseline and 15 year follow-up.

Loneliness also appeared to moderate the impact of ILI and DSE on mobility. The interaction indicated that the long-term effect of ILI on gait speed and the 400 m walk test did not differ by loneliness. However, in DSE, loneliness related to poorer outcomes for gait speed and the 400 m walk test, leading to treatment arm by loneliness interactions. This suggests that ILI benefited mobility regardless of loneliness level, whereas DSE predicted a lesser overall benefit and the benefit varied by loneliness. As DSE had minimal intervention, it is plausible that the relationship of loneliness to mobility in DSE is reflective of prior reports of associations with health outcome in more general populations, e.g., [15–17]. However, the impact of ILI on mobility may have blunted relationships with loneliness.

It further appeared that ILI was more effective in reducing HbA1c compared with DSE among individuals with higher loneliness, whereas there was little difference by treatment arm among those with lower loneliness levels. It is plausible that ILI may have been more effective among individuals with higher loneliness scores than DSE due to the differential contacts with providers and other participants in ILI. However, it should be recognized that the interaction analyses were exploratory and not hypothesis driven.

Taken together, these findings from a sample of older participants who have type 2 diabetes and overweight or obesity share many similarities to the prior literature involving older people without diabetes. Specifically, we find loneliness to relate to disability scores, objective gait speed, depressive symptoms and use of antidepressant medications, and health-related quality of life, consistent with prior reports [5, 7, 13, 15–18]. In contrast, we did not support prior studies finding relationships of loneliness with glycemic control. Given that diabetes impacts one in four elderly adults in the United States, requires a complicated self-management regimen, and portends an increased burden from multiple diseases, it is critical to identify factors that may further compound disease risk.

One challenge of the literature relating loneliness to health outcomes is the variety of different scales used. Meta-analysis and reviews, e.g., [1, 4], identify numerous scales designed to characterize loneliness, with some scales showing overlap with structural measures of social connection. Some of the prior research has even relied on a single question to index loneliness. We used the UCLA Brief Loneliness Scale (three items). This scale shows a strong correspondence with the original, 20-item questionnaire [29] and has previously been demonstrated to relate to behavioral and physical outcomes, including depression [7], physical function [16], and mortality [9], among other outcomes. Thus, the scale was a reasonable choice to represent the health impacts of loneliness.

It is important to note that the present study did not measure social isolation, a related but distinct measure of the structure of social contacts. Social isolation also predicts aging-related outcomes [9, 12]. Indeed, several research studies have compared the impact of loneliness to social isolation to determine whether the perception or structure of social contacts has a greater impact on health, but research remains mixed [1, 6, 9, 15, 16, 23, 35]. It is also plausible that loneliness and social isolation have synergistic effects wherein those with both conditions are at the greatest health risk [36]. Future research in Look AHEAD should incorporate a measure of social isolation, in addition to loneliness, to determine the relative contributions of each and the potential for compounding risks.

Given the health risks, it is encouraging that interventions are being tested to reduce loneliness in the elderly. For example, Silver Sneakers, a gym membership and exercise classes, was shown to increase physical activity and to reduce social isolation and loneliness compared to matched controls [37]. In addition, a pilot, m-Health intervention targeting maladaptive cognitions in elderly individuals who are experiencing loneliness reduced these symptoms over three months [38].

Although this study had several strengths, including an aging sample with type 2 diabetes, a large sample size, and objective measures of physical function, it is important to note limitations. First, as this study was cross-sectional, the direction of association cannot be determined. Indeed, prior research suggests that the associations of loneliness with depression and disability may be bidirectional [7, 18]. Future, longitudinal research will be needed to resolve directionality in this cohort. In addition, the loneliness questionnaire was only included in the clinic questionnaire, which excluded 9.4% of participants who completed telephone interviews but may have had more difficulty attending visits in the clinic. Neither social isolation nor contemporaneous socioeconomic status was measured in Look AHEAD and may serve as confounders of the relationship between loneliness and the health outcomes. Lastly, although we did not see evidence for differential loss of follow-up between treatment groups, we cannot rule out that this may have introduced bias in differences and associations.

Overall, this study demonstrates that loneliness relates to greater disability, slower gait speed, depressive symptoms, antidepressant medication use, and poorer quality of life among older individuals who have type 2 diabetes and are overweight or obese. Future longitudinal research needs to address questions such as the potential for bidirectional relationships with loneliness and the longer-term impacts on health outcomes.

## Figures and Tables

**Table 1:** Characteristics of participants with nonmissing loneliness scale at the Look AHEAD-E visit.

	Nonmissing	Overall	Intervention arm		*p* value
			DSE	ILI	
*N*		3190	1553	1634	
Baseline characteristics					
Age	3190	58.3 (6.4)	58.3 (6.4)	58.2 (6.3)	0.7042
BMI	3190	35.9 (6.0)	36.0 (5.8)	35.7 (6.1)	0.1338
Gender	3190				0.4756
Male		1214 (38.1%)	581 (37.4%)	633 (38.7%)	
Female		1976 (61.9%)	973 (62.6%)	1003 (61.4%)	
Race/ethnicity	3190				0.6896
White		1939 (60.8%)	950 (61.1%)	989 (60.5%)	
Black		524 (16.4%)	260 (16.7%)	264 (16.2%)	
Hispanic		440 (13.8%)	203 (13.1%)	237 (14.5%)	
Others		287 (9.0%)	142 (9.1%)	145 (8.9%)	
LA-E visit					
Age	3190	72.7 (6.2)	72.7 (6.3)	72.7 (6.1)	0.7424
BMI	3019	33.2 (6.2)	33.6 (6.2)	32.9 (6.1)	0.0012
HbA1c%	2665	7.5 (1.5)	7.5 (1.5)	7.5 (1.4)	0.5779
PHQ-9	3052	2.7 (3.3)	2.8 (3.4)	2.6 (3.2)	0.1786
PHQ-9 = 0	3052	937 (30.7%)	458 (30.8%)	479 (30.6%)	0.9079
SF-36 general health	3156	2.9 (0.8)	2.9 (0.8)	2.9 (0.8)	0.4558
PAT-D	3157	1.5 (0.5)	1.5 (0.5)	1.5 (0.5)	0.5800
PAT-D = 1	3157	419 (13.3%)	212 (13.8%)	207 (12.8%)	0.4165
400 m walk time (min)	2632	6.7 (1.9)	6.8 (2.0)	6.7 (1.9)	0.6073
Gait speed test (sec)	2949	4.93 (1.67)	5.00 (1.71)	4.85 (1.62)	0.0168
Grip strength (right hand)	2702	23.9 (9.4)	23.8 (9.5)	24.0 (9.3)	0.5225
Taking antidepressants	2822	699 (24.8%)	337 (24.5%)	362 (25.0%)	0.7546
Taking any diabetes med	3067	2814 (91.8%)	1380 (92.3%)	1434 (91.2%)	0.2744
Biguanide	2967	2063 (69.5%)	1008 (69.5%)	1055 (69.6%)	0.9868
Insulin	2894	1344 (46.4%)	704 (49.5%)	640 (43.5%)	0.0010
Sulfonylurea	2863	979 (34.2%)	490 (35.1%)	489 (33.4%)	0.3460
TZD	2758	198 (7.2%)	94 (7.0%)	104 (7.4%)	0.6900
Loneliness	3190	3.86 (1.38)	3.90 (1.42)	3.82 (1.34)	0.1164

Values are given as mean (SD) or *N* (%)

**Table 2:** Adjusted associations with loneliness.

Outcome	Model 1			Model 2			Model 3		
	Beta	SE	*p* value	Beta	SE	*p* value	Beta	SE	*p* value

Gait speed test (seconds)	0.192	0.022	<0.0001	0.097	0.026	0.0002	0.096	0.026	0.0003
Grip strength (right hand)	−0.382	0.097	<0.0001	−0.170	0.117	0.1471	−0.168	0.118	0.1530
400 m walk time	0.034	0.029	0.2451	0.038	0.035	0.2759	0.037	0.035	0.2839
SF-36 general health	0.140	0.011	<0.0001	0.035	0.012	0.0057	0.035	0.013	0.0058
HbA1c%	0.030	0.020	0.1372	0.009	0.023	0.6868	0.010	0.023	0.6632

	OR	95% CI	*p* value	OR	95% CI	*p* value	OR	95% CI	*p* value

PAT-D > 1	1.470	1.313–1.645	<0.0001	1.243	1.085–1.424	0.0017	1.245	1.087–0.427	0.0016
PHQ-9 > 0	1.897	1.723–2.088	<0.0001	1.752	1.585–1.938	<0.0001	1.754	1.586–1.939	<0.0001
Taking antidepressants	1.319	1.242–1.401	<0.0001	1.126	1.048–1.211	0.0013	1.127	1.048–1.211	0.0012

*Note.* Model 1: adjusts for age, sex, and race/ethnicity. Model 2: Model 1 plus depressive symptoms (PHQ-9) and antidepressant medication use. Also includes major diabetes med categories for HbA1c outcome (biguanide, insulin, sulfonylurea, and TZD) PHQ-9 outcome does not adjust for depressive symptoms. Taking antidepressants outcome does not adjust for antidepressant medication use. Model 3: Model 2 plus treatment arm.

**Table 3:** Exploring interactions with loneliness.

Outcome	Loneliness by treatment arm (*p* value)
Gait speed test (seconds)	**0.0325**

Grip strength (right hand)	0.0568

400 m walk time	**0.0253**

HbA1c%	**0.0441**

SF-36 general health	0.2164

PAT-D > 1	0.1308
PHQ-9 > 0	0.2819
Taking antidepressants	0.8536

## Data Availability

All Look AHEAD data will be made available through the National Institute of Diabetes, Digestive, and Kidney Disease data repository within two years in accordance with the policy of the Look AHEAD clinical trial.

## Supplementary Materials

Supplementary Materials Supplementary Table 1: levels of loneliness by participant characteristics. Supplementary Figure 1(a): interaction between loneliness score and treatment arm in relation to 400 meter walk time. Interaction illustrated using the 10^th^ (loneliness = 3) and 90^th^ (loneliness = 6) percentiles for the loneliness score. Supplementary Figure 1(b): interaction between loneliness score and treatment arm in relation to gait speed. Interaction illustrated using the 10^th^ (loneliness = 3) and 90^th^ (loneliness = 6) percentiles for the loneliness score. Supplementary Figure 1(c): interaction between loneliness score and treatment arm in relation to hemoglobin A1c. Interaction illustrated using the 10^th^ (loneliness = 3) and 90^th^ (loneliness = 6) percentiles for the loneliness score.
