# Supplementary material for: Loneliness Relates to Functional Mobility in Older Adults with Type 2 Diabetes: The Look AHEAD Study
Source: J Aging Res. 2020 Oct 30;2020:7543702. doi: 10.1155/2020/7543702 (PMC7647748; doi:10.1155/2020/7543702)
Supplement: Supplementary Materials — Supplementary Table 1: levels of loneliness by participant characteristics. Supplementary Figure 1(a): interaction between loneliness score and treatment arm in relation to 400 meter walk time. Interaction illustrated using the 10th (loneliness = 3) and 90th (loneliness = 6) percentiles for the loneliness score. Supplementary Figure 1(b): interaction between loneliness score and treatment arm in relation to gait speed. Interaction illustrated using the 10th (loneliness = 3) and 90th (loneliness = 6) percentiles for the loneliness score. Supplementary Figure 1(c): interaction between loneliness score and treatment arm in relation to hemoglobin A1c. Interaction illustrated using the 10th (loneliness = 3) and 90th (loneliness = 6) percentiles for the loneliness score. [file 7543702.f1.zip › 7543702.f1/Supplemental_fig_1c.docx]

Supplemental Figure 1c
