# Supplementary material for: Loneliness Relates to Functional Mobility in Older Adults with Type 2 Diabetes: The Look AHEAD Study
Source: J Aging Res. 2020 Oct 30;2020:7543702. doi: 10.1155/2020/7543702 (PMC7647748; doi:10.1155/2020/7543702)
Supplement: Supplementary Materials — Supplementary Table 1: levels of loneliness by participant characteristics. Supplementary Figure 1(a): interaction between loneliness score and treatment arm in relation to 400 meter walk time. Interaction illustrated using the 10th (loneliness = 3) and 90th (loneliness = 6) percentiles for the loneliness score. Supplementary Figure 1(b): interaction between loneliness score and treatment arm in relation to gait speed. Interaction illustrated using the 10th (loneliness = 3) and 90th (loneliness = 6) percentiles for the loneliness score. Supplementary Figure 1(c): interaction between loneliness score and treatment arm in relation to hemoglobin A1c. Interaction illustrated using the 10th (loneliness = 3) and 90th (loneliness = 6) percentiles for the loneliness score. [file 7543702.f1.zip › 7543702.f1/Supplementalfigure1a.docx]

Supplemental Figure 1a

Supplementary Table 1: Levels of loneliness by participant characteristics

|  | **Loneliness** | | | |
| --- | --- | --- | --- | --- |
|  | **N** | **Mean** | **SD** | **ANOVA**  **p-value** |
| Overall | 3190 | 3.86 | 1.38 |  |
| Baseline Age |  |  |  | 0.1615 |
| 45-54 | 839 | 3.93 | 1.40 |  |
| 55-64 | 1883 | 3.82 | 1.37 |  |
| 65+ | 468 | 3.88 | 1.39 |  |
| Gender |  |  |  | <.0001 |
| Male | 1214 | 3.73 | 1.31 |  |
| Female | 1976 | 3.94 | 1.42 |  |
| Race/Ethnicity |  |  |  | 0.0001 |
| White | 1939 | 3.78 | 1.33 |  |
| Black | 524 | 3.90 | 1.38 |  |
| Hispanic | 440 | 4.07 | 1.52 |  |
| Other | 287 | 4.01 | 1.42 |  |
| Education |  |  |  | <.0001 |
| HS or less | 622 | 4.03 | 1.50 |  |
| Some college | 1154 | 3.93 | 1.43 |  |
| Bachelors or more | 1329 | 3.72 | 1.26 |  |
| Baseline BMI |  |  |  | 0.0182 |
| <30 | 500 | 3.84 | 1.33 |  |
| 30-40 | 1982 | 3.82 | 1.34 |  |
| 40+ | 708 | 3.99 | 1.52 |  |
| Age at LA-E Visit |  |  |  | 0.3842 |
| 55-64 | 413 | 3.93 | 1.36 |  |
| 65-74 | 1707 | 3.83 | 1.36 |  |
| 75+ | 1070 | 3.87 | 1.42 |  |
| BMI at LA-E Visit |  |  |  | <.0001 |
| <30 | 928 | 3.80 | 1.31 |  |
| 30-40 | 1586 | 3.79 | 1.30 |  |
| 40+ | 407 | 4.12 | 1.66 |  |
